# Unveiling the Enigmatic Role of SLC35F3 in Lung Adenocarcinoma

**DOI:** 10.1111/crj.70023

**Published:** 2024-10-16

**Authors:** Yiwang Ye, Feihu Long, Wei Yue, Zichun Wei, Jianyi Yang, Yuancai Xie

**Affiliations:** ^1^ Department of Thoracic Surgery Peking University Shenzhen Hospital Shenzhen China

**Keywords:** drug sensitivity, immune infiltration, lung adenocarcinoma, prognosis, solute carrier family 35 member F3, TMB

## Abstract

**Background:**

The role of solute carrier family 35 member F3 (SLC35F3) in lung adenocarcinoma (LUAD) remains unclear. To address this gap, we conducted a study employing bioinformatics analysis and experimental validation.

**Methods:**

This study aimed to examine the expression patterns of SLC35F3 in various cancer types, particularly focusing on LUAD, by analyzing data from the Cancer Genome Atlas (TCGA) database to evaluate its clinical relevance. The research also explored potential regulatory mechanisms of SLC35F3, including its interactions with immune infiltration, tumor mutational burden (TMB), and drug sensitivity in LUAD. The investigation included analyzing SLC35F3 expression in single‐cell sequencing of LUAD cells, examining genetic variations of SLC35F3 in LUAD, and assessing SLC35F3 expression in cell lines using quantitative real‐time PCR (qRT‐PCR).

**Results:**

The aberrant expression of SLC35F3 was observed in both pan‐cancer and LUAD. In LUAD patients, a statistically significant increase in SLC35F3 expression was correlated with gender (*p* < 0.001) and was associated with poorer overall survival (OS) (*p* = 0.020). The expression of SLC35F3 was identified as an independent prognostic determinant in patients with LUAD (*p* = 0.032). SLC35F3 exhibited associations with various pathways, including cell cycle and more. SLC35F3 expression demonstrated correlations with immune infiltration, TMB, and some drugs in LUAD. Results indicated significant upregulation of SLC35F3 in both LUAD tissues and cell lines.

**Conclusions:**

SLC35F3 may serve as a prognostic biomarker and immunotherapeutic target for patients with LUAD.

**Clinical Trial Registration:**

Not applicable.

## Graphical abstract

Increased expression of SLC35F3 in LUAD patients was significantly associated with poorer overall survival (OS) (*p* = 0.020). The expression of SLC35F3 was identified as an independent prognostic determinant in patients with LUAD (*p* = 0.032). SLC35F3 may serve as a promising prognostic biomarker and therapeutic target for LUAD patients.

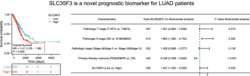

## 1 Introduction

Non‐small cell lung cancer (NSCLC) is a widespread malignancy on a global scale, contributing significantly to cancer‐related mortality [1]. Lung adenocarcinoma (LUAD) has emerged as the predominant subtype of NSCLC and a major contributor to cancer‐related mortality on a global scale [2]. The 5‐year survival rate of NSCLC is largely dependent on the disease stage, with survival rates varying from around 80% in Stage I to as low as 10% in cases of metastatic disease [3]. Surgical resection is the established treatment for early‐stage NSCLC. The management of advanced or metastatic disease has seen significant progress in recent years, particularly because of advancements in molecular targeted therapy and immunotherapy [4, 5, 6]. However, because of the limitations of targeted therapy for LUAD with driver mutations and variable immune therapy responses, only a few patients benefit from these treatments [7]. The pathogenesis of LUAD is a complex process that is closely associated with abnormal gene expression [8]. Consequently, a comprehensive understanding of the molecular underpinnings of LUAD could yield more precise biomarkers for tumor diagnosis and management.

The solute carrier family member F3 (SLC35F3) is a transporter that absorbs thiamine through cell and mitochondrial membranes [9]. SLC35F3 is associated with the risk of hypertension [9, 10]. The interaction between SLC35F3 and dietary carbohydrate intake has a significant impact on the incidence rate of metabolic syndrome (MetS) [11]. SLC35F3 is associated with the diversity of skeletal progenitor cells [12]. The diagnostic model based on TPRC5, TINM1, NELL2, DMD, SLC35F3, and AT2 has a good effect in distinguishing papillary thyroid carcinoma (PTC) from normal tissue [13]. However, the exact role of SLC35F3 in LUAD is not yet fully understood.

This study aimed to analyze the expression levels of SLC35F3 in different cancer types, particularly focusing on LUAD, by utilizing data from the Cancer Genome Atlas (TCGA) database to evaluate its potential diagnostic significance [14, 15]. LUAD clinical features and prognosis were also examined in relation to SLC35F3 expression levels. The research explored the potential regulatory mechanisms associated with SLC35F3, including its interactions with immune infiltration, tumor mutational burden (TMB), and drug sensitivity in LUAD [16]. The study also encompassed an examination of SLC35F3 expression in single‐cell sequencing of LUAD cells, genetic variations of SLC35F3 in LUAD, and SLC35F3 expression in cell lines using quantitative real‐time PCR (qRT‐PCR). The findings suggest that targeting SLC35F3 could potentially offer therapeutic and prognostic benefits to individuals with LUAD.

## 2 Materials and Methods

### 2.1 Expression of SLC35F3 in Pan‐Cancer and TCGA‐LUAD

A cohort comprising 539 patients diagnosed with LUAD and 59 normal tissue samples were sourced from the TCGA dataset [17]. The specific molecule of interest was SLC35F3 [ENSG00000183780.13]. RNA sequencing data in transcript per million (TPM) format from TCGA and GTEx projects, processed consistently through the Toil pipeline, were accessed through UCSC XENA and the TCGA portal. Pan‐cancer data from TCGA and GTEx normal tissue samples were collected for subsequent analysis. The data were processed in log2 (value + 1). Data visualization was performed using the ggplot2 package, and the choice of statistical methods (stats package and car package) was based on the characteristics of the data format.

RNAseq data from the TCGA database were retrieved and processed using the STAR method within the TCGA‐LUAD project and subsequently converted into TPM format. Data were then processed using the log2 (value + 1) transformation.

### 2.2 SLC35F3 and Its Diagnostic Value Related to Clinical Characteristics

We used software version R (4.2.1) for the statistics. The data filtering strategy was to remove normal and nonclinical information.

To visualize the results, ggplot2 was used to perform a ROC analysis on the data [18].

### 2.3 The Relationship Between SLC35F3 and Prognosis

The survival package was used for testing proportional risk hypothesis and fitting survival regressions, as well as the ggplot2 package for visualization. The prognosis included overall survival (OS).

Forest plots were created using R software (version 3.6.3) with the ggplot2 package.

The nomogram plots were plotted with both the rms and survival packages. Variables including T stage, N stage, pathological stage, and primary therapy outcome were considered in determining the type of prognosis [19].

### 2.4 Gene Set Enrichment Analysis (GSEA)

ClusterProfiler (version 4.4.4) and R (version 4.2.1) were utilized for further analysis. Following molecular identification conversion, GSEA was conducted using the clusterProfiler package, focusing on human ( *Homo sapiens* ) species. The sets of genes used for reference were c2.cp.all.v2022.1.Hs.symbols.gmt (3050).

### 2.5 The Correlation Between SLC35F3 and Immune Infiltration

As a result of calculating the immune infiltration of the corresponding cloud data using markers provided in the reference, the GSVA R package [1.46.0] provided the ssGSEA algorithm.

### 2.6 The Relationship Between SLC35F3 and TMB and SLC35F3 Expression in Single Cells of LUAD

TMB is employed for quantifying the mutational load in cancer [20, 21, 22]. In LUAD, SLC35F3 expression was explored using TISH2 (http://tisch.comp‐genomics.org/ [23].

### 2.7 Analysis of SLC35F3 Mutations in LUAD Patients

Level 3 RNAseq data, mutation maf data, and pertinent clinical data for LUAD were extracted from the TCGA dataset [14, 22]. The maftools package within the R software environment was employed for the retrieval and visualization of somatic mutations in LUAD patients [24, 25]. Visual representation in the form of horizontal histograms demonstrated a notable prevalence of mutations among LUAD patients [22].

### 2.8 The Relationship Between SLC35F3 and Response to Drug Treatment

The RNAactDrug database, accessible at http://bio‐bigdata.hrbmu.edu.cn/RNAactDrug/index.jsp, was utilized to evaluate the drug sensitivity of SLC35F3 across various cancer types [26, 27].

### 2.9 Validation of SLC35F3 Expression in GSE87340 and Cell Lines

GSE87340 was used to validate the expression of SLC35F3 in LUAD. GSE74706 included 28 tumor samples and 26 normal samples.

BEAS‐2B, A549, NCI‐H1299, and HCC827 cells were obtained from the Chinese Academy of Sciences and cultured in high glucose DMEM medium with 10% fetal bovine serum (FBS) and 1% penicillin–streptomycin at 37°C in a 5% CO_2_ environment [28]. The expression levels of SLC35F3 were quantified through qRT‐PCR in the aforementioned cell lines, in accordance with established protocols outlined in the literature. The primer sequences employed for the qRT‐PCR analysis are provided below [13]:

GAPDH‐F: 5′‐ACAACTTTGGTATCGTGGAAGG‐3′;
GAPDH‐R: 5′‐GCCATCACGCCACAGTTTC‐3′;
SLC35F3‐F: 5′‐CGGGCGCAACTCAAGAAGAT‐3′;
SLC35F3‐R: 5′‐CCCACGTAGTACAACGGGA‐3′.

### 2.10 Statistical Analysis

The aforementioned analytical methods and R packages were implemented utilizing R software version 4.0.3 (R Foundation for Statistical Computing, 2020). The statistical methodologies employed encompassed the Wilcoxon signed rank test and *t*‐test, with statistical significance established at *p*‐values below 0.05 [29].

## 3 Results

### 3.1 SLC35F3 Was Differentially Expressed in LUAD and Some Tumors

**FIGURE 1:**
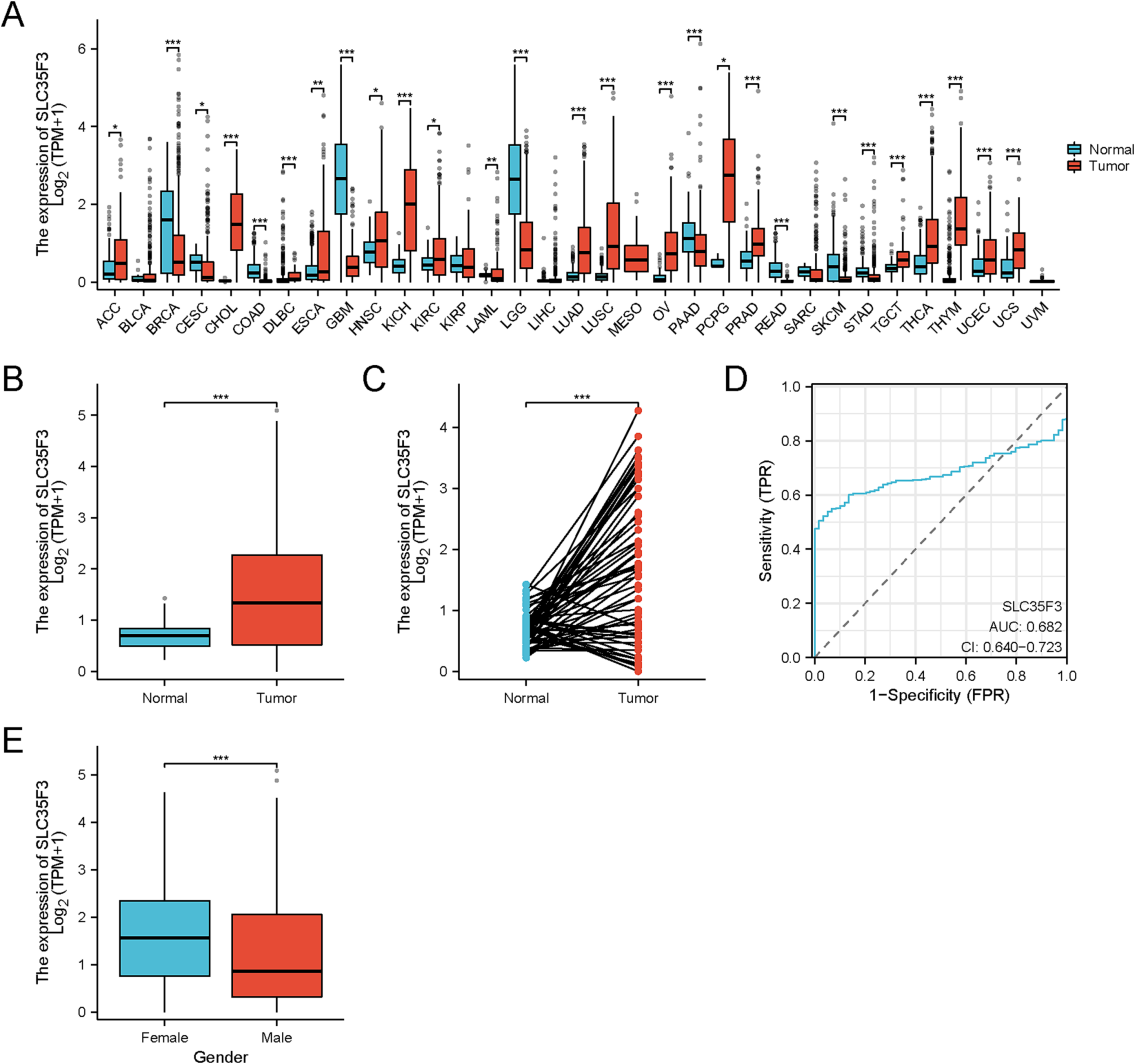
Aberrant SLC35F3 expression in LUAD correlates with clinical features. (A) Pan‐cancer (TCGA + GTEx) expresses significantly more SLC35F3. (B) Unpaired samples. (C) Paired samples. (D) LUAD and normal lung tissues were distinguished by SLC35F3 expression levels. (E) Gender. * *p* < 0.05; ** *p* < 0.01; *** *p* < 0.001.

The clinical characteristics of TCGA‐LUAD were collected and analyzed in this study, as presented in Table S1. A comprehensive pan‐cancer analysis of SLC35F3 expression was undertaken, as illustrated in Figure 1A, unveiling notable upregulation in 18 tumors and significant downregulation in nine tumors. Consequently, it can be concluded that aberrant expression of SLC35F3 is associated with tumor development. Based on the data presented in Figure 1B–C, the expression level of SLC35F3 in LUAD tumor tissues exhibited a significant increase compared with both normal unpaired tissues (*p* < 0.001) and paired normal tissues (*p* < 0.001). Furthermore, Figure 1D illustrates that the area under the curve (AUC) of SLC35F3 was calculated to be 0.682, indicating its potential utility as a valuable biomarker for distinguishing LUAD from normal lung tissues.

### 3.2 SLC35F3 Was Significantly Associated With Clinical Features in LUAD

The results presented in Table S2 and Figure 1E demonstrate a significant correlation between elevated SLC35F3 expression in patients with LUAD and gender (*p* < 0.001).

### 3.3 A Significant Association Between SLC35F3 Expression and Prognosis in LUAD

**FIGURE 2:**
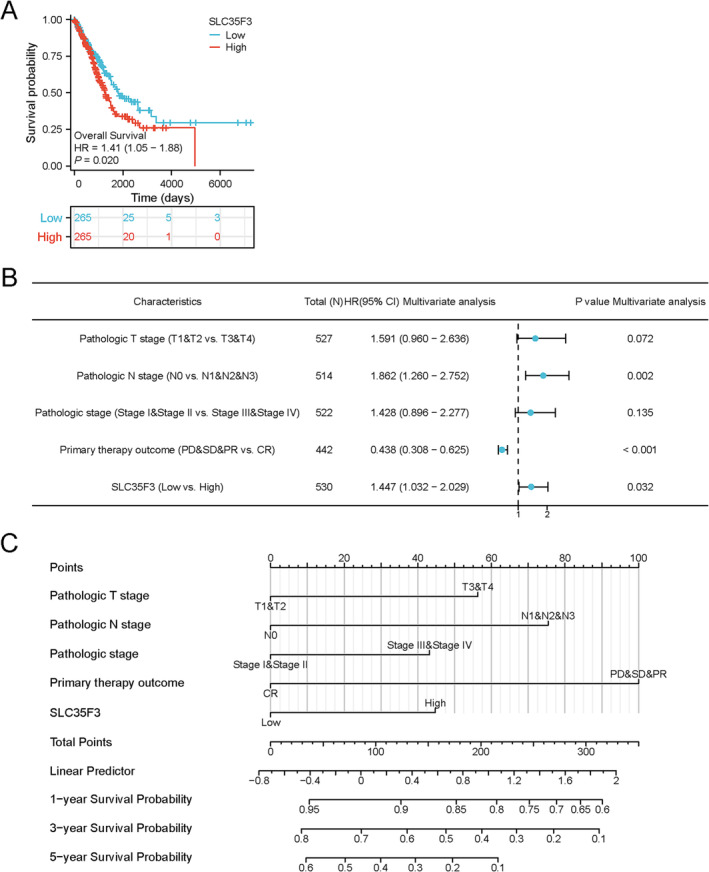
LUAD patients with high expression of SLC35F3 have poor OS. (A) OS. (B) Analysis of multivariate Cox regression in LUAD by forest plot. (C) Using a nomogram to predict OS probability over 1, 3, and 5 years.

Patients with high expression of SLC35F3 exhibited a poorer OS (*p* = 0.020), suggesting the potential of SLC35F3 as a prognostic gene in predicting negative outcomes in LUAD and providing valuable insights for future treatment strategies. Univariate Cox regression analysis revealed that factors such as T stage (*p* < 0.001), N stage (*p* < 0.001), pathologic stage (*p* < 0.001), primary outcome of therapy (*p* < 0.001), and SLC35F3 (*p* = 0.02) were significantly correlated with OS in LUAD patients, as shown in Table S3 and Figure 2B.

The results of the multivariate Cox regression analysis indicated significant associations between the N stage (*p* = 0.002), primary therapy outcome (*p* < 0.001), and SLC35F3 (*p* = 0.032) with the OS of patients diagnosed with LUAD, as presented in Table S3 and Figure 2B. SLC35F3 was identified as an independent prognostic factor that influences the outcome of patients with LUAD. The use of column line graphs, as shown in Figure 2C, facilitated the estimation of 1‐, 3‐, and 5‐year survival probabilities for patients with LUAD based on the expression of SLC35F3 and relevant clinical variables.

### 3.4 Pathways Involved in SLC35F3 in LUAD

**FIGURE 3:**
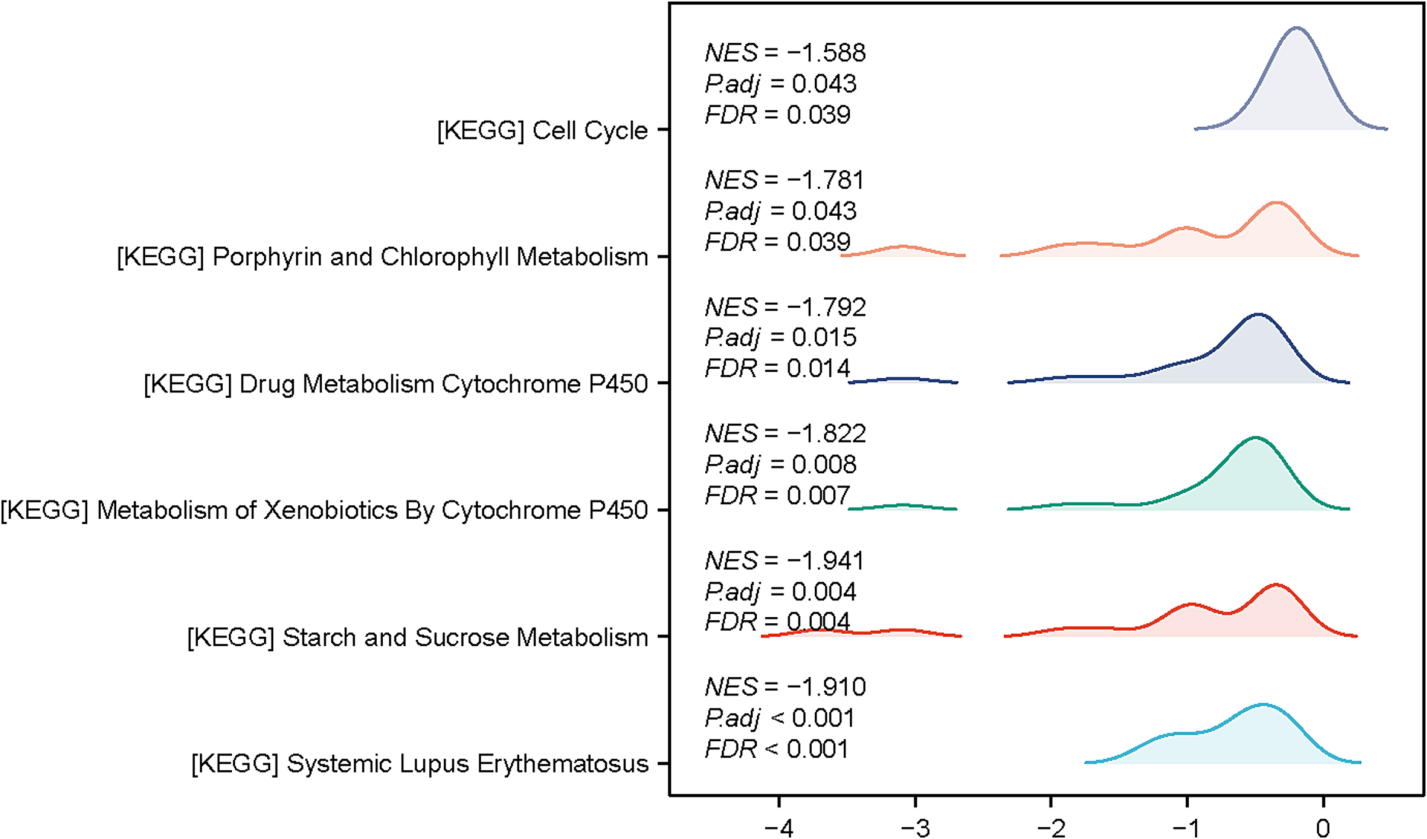
Analysis of gene set enrichment (GSEA) enrichment plots.

In order to explore the potential mechanism of SLC35F3 in LUAD, a collection of SLC35F3‐associated genes was identified through GSEA analysis. Among these genes, nine pathways were determined to have significant associations with SLC35F3, including systemic lupus erythematosus (SLE), starch and sucrose metabolism, metabolism of xenobiotics by cytochrome p450, drug metabolism cytochrome p450, porphyrin and chlorophyll metabolism, and cell cycle (Figure 3).

### 3.5 SLC35F3 Was Significantly Associated With Immune Infiltration and TMB

**FIGURE 4:**
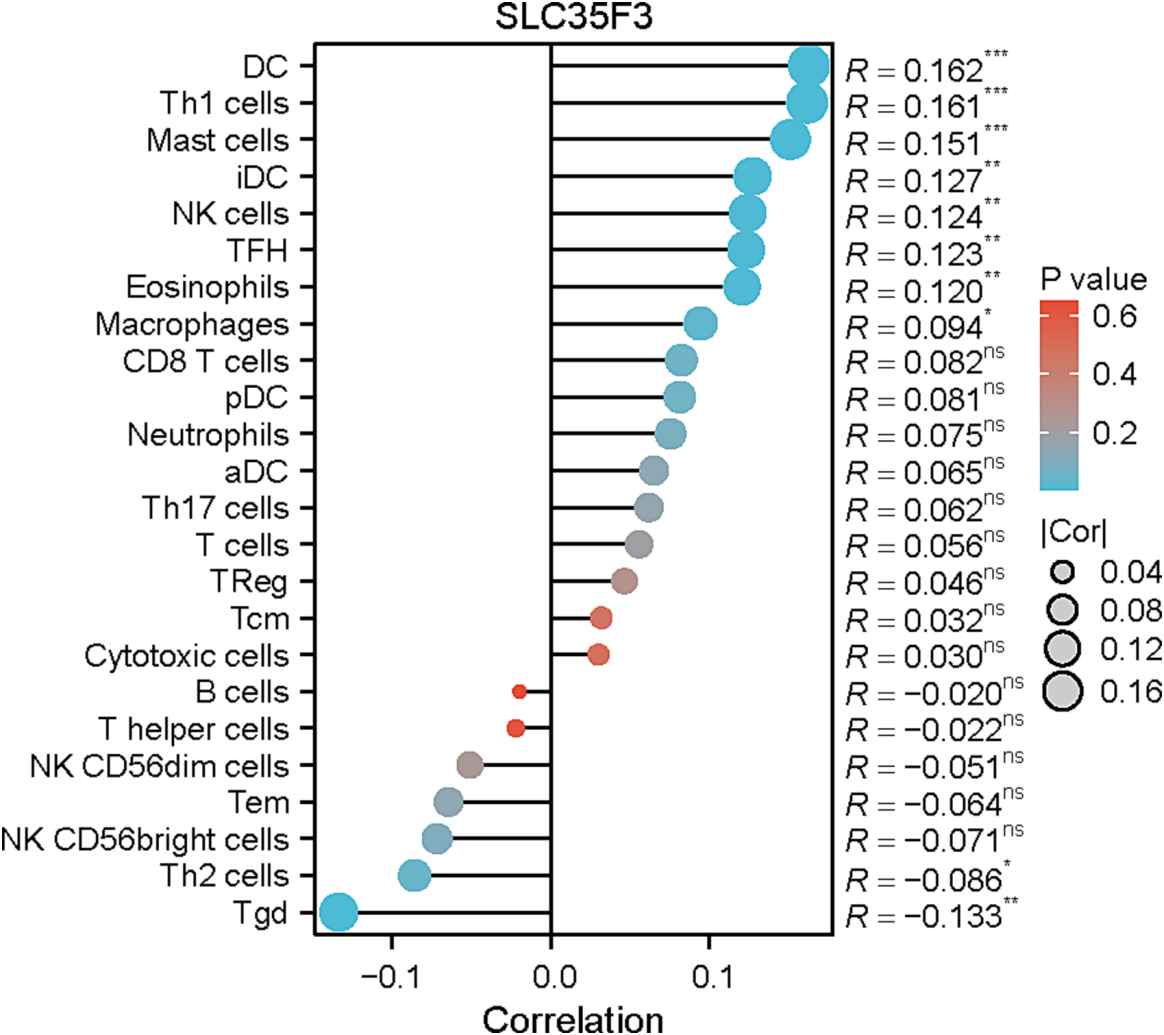
SLC35F3 expression was associated with immune infiltration in LUAD. ns, *p* > 0.05; * *p* < 0.05; ** *p* < 0.01; *** *p* < 0.001.

**FIGURE 5:**
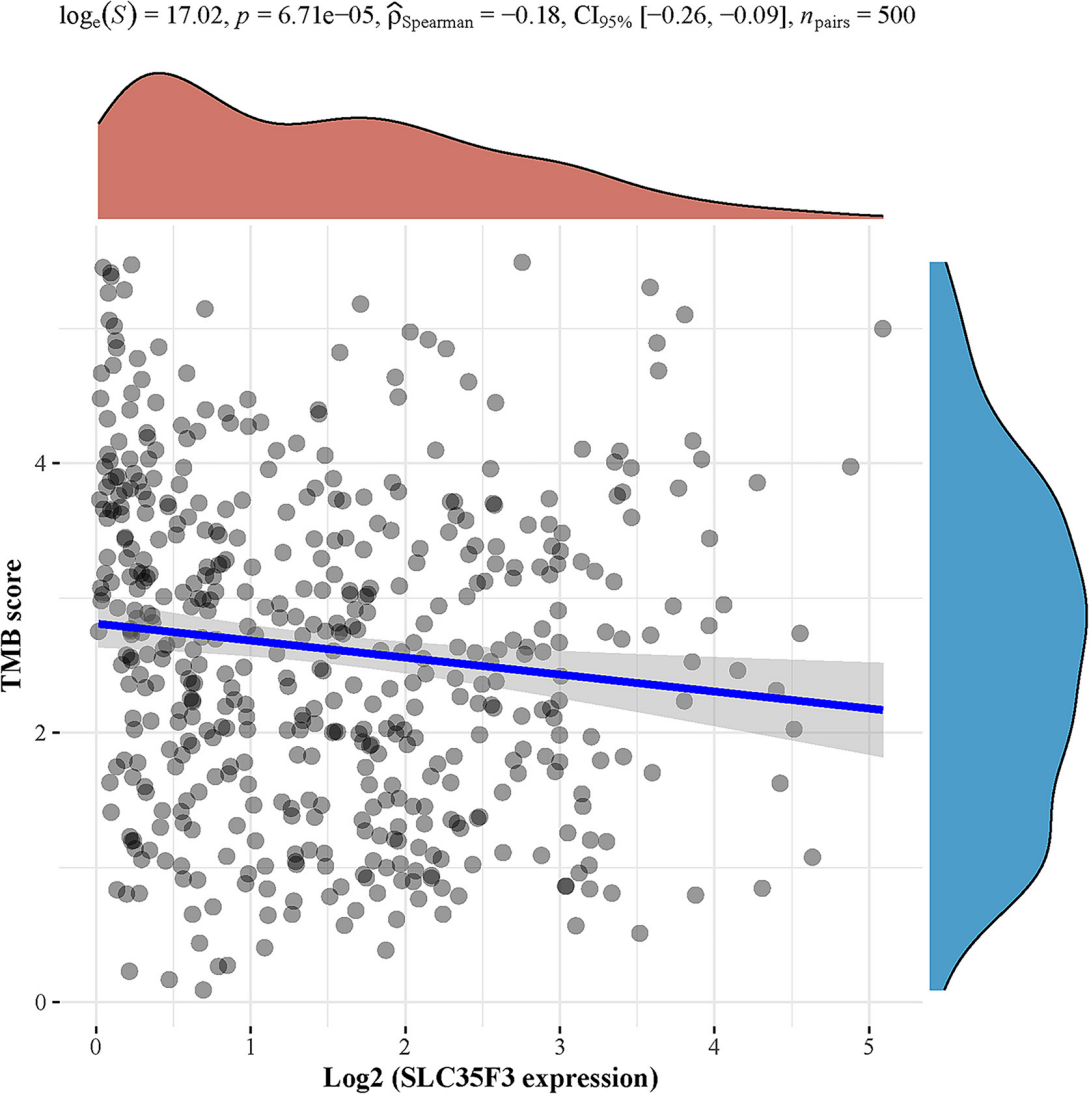
LUAD exhibited SLC35F3 expression associated with TMB.

Figure 4 demonstrates a statistically significant positive correlation between the expression levels of SLC35F3 and various immune cell types, including DC (*p* < 0.001), eosinophils (*p* = 0.005), iDC (*p* = 0.003), macrophages (*p* = 0.029), mast cells (*p* < 0.001), NK cells (*p* = 0.004), TFH (*p* = 0.004), and Th1 cells (*p* < 0.001). Furthermore, a significant negative correlation was observed between the expression of SLC35F3 and Tgd (*p* = 0.002) and Th2 cells (*p* = 0.047). In LUAD, a significant negative correlation exists between the expression of SLC35F3 and MSI (*p* = 6.71e‐05, as illustrated in Figure 5).

### 3.6 SLC35F3 Is Significantly Upregulated in LUAD Single Cells

**FIGURE 6:**
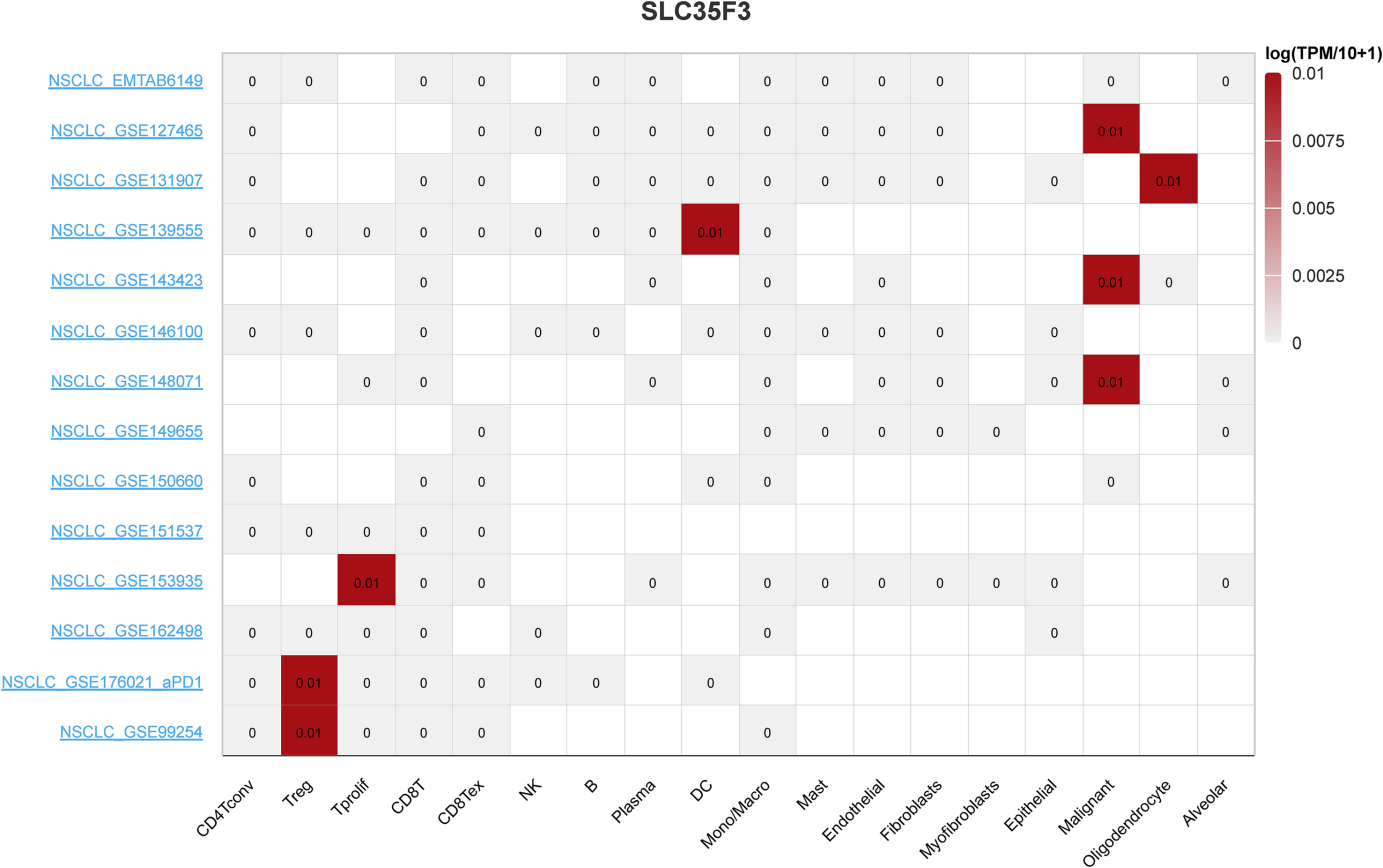
Single cells from LUAD were found to express SLC35F3.

As shown in Figure 6, the SLC35F3 gene was upregulated in multiple individual cells of LUAD, including Treg, Tprolif, DC, Malignant, and Oligodendrocyte.

### 3.7 Somatic Variants of SLC35F3 in LUAD

**FIGURE 7:**
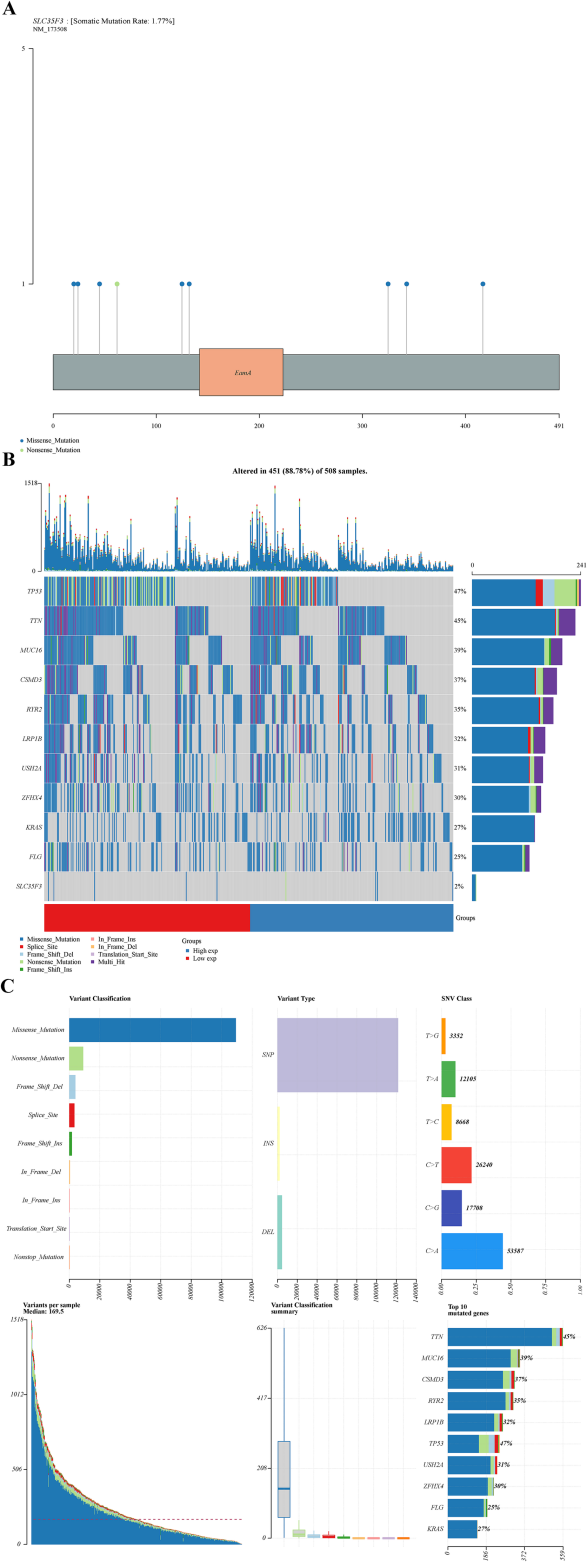
Somatic mutations of SLC35F3 in LUAD. (A) Lollipop plot showing the distribution of SLC35F3 gene mutations. (B) The oncoplot shows the somatic landscape of SLC35F3. (C) The cluster summary plots show how variants are classified, types, and SNVs are categorized.

The results presented in Figure 7A indicate that somatic variants of SLC35F3 in LUAD comprised eight missense mutations and one nonsense mutation. The somatic mutation rate of SLC35F3 in LUAD was determined to be 1.77%. Analysis of the top 10 mutated genes in both high‐ and low‐expression groups of SLC35F3 revealed the presence of TP53 (47%), TTN (45%), MUC16 (39%), CSMD3 (37%), RYR2 (35%), LRP1B (32%), USH2A (31%), ZFHX4 (30%), KRAS (27%), and FLG (25%), as depicted in Figure 7B. Figure 7C highlights that missense mutations were the predominant variant classification, with SNPs, particularly the C > A mutation, being the most commonly observed.

### 3.8 SLC35F3 Expression Correlates With Drug Sensitivity

The present study utilized the RNAactDrug database to investigate the potential relationship between SLC35F3 expression levels and drug responsiveness. The results revealed a statistically significant positive correlation between SLC35F3 expression and the effectiveness of formycin a, panobinostat, CCT007093, phenformin, and selisistat (Table S4). Conversely, the findings also indicated a negative correlation between SLC35F3 expression and the sensitivity to 2‐bromo‐, holacanthone, gw694234a, and more (Table S4). These results suggest a possible connection between SLC35F3 and drug resistance to specific medications.

### 3.9 SLC35F3 Is Aberrantly Expressed in LUAD Tissues

**FIGURE 8:**
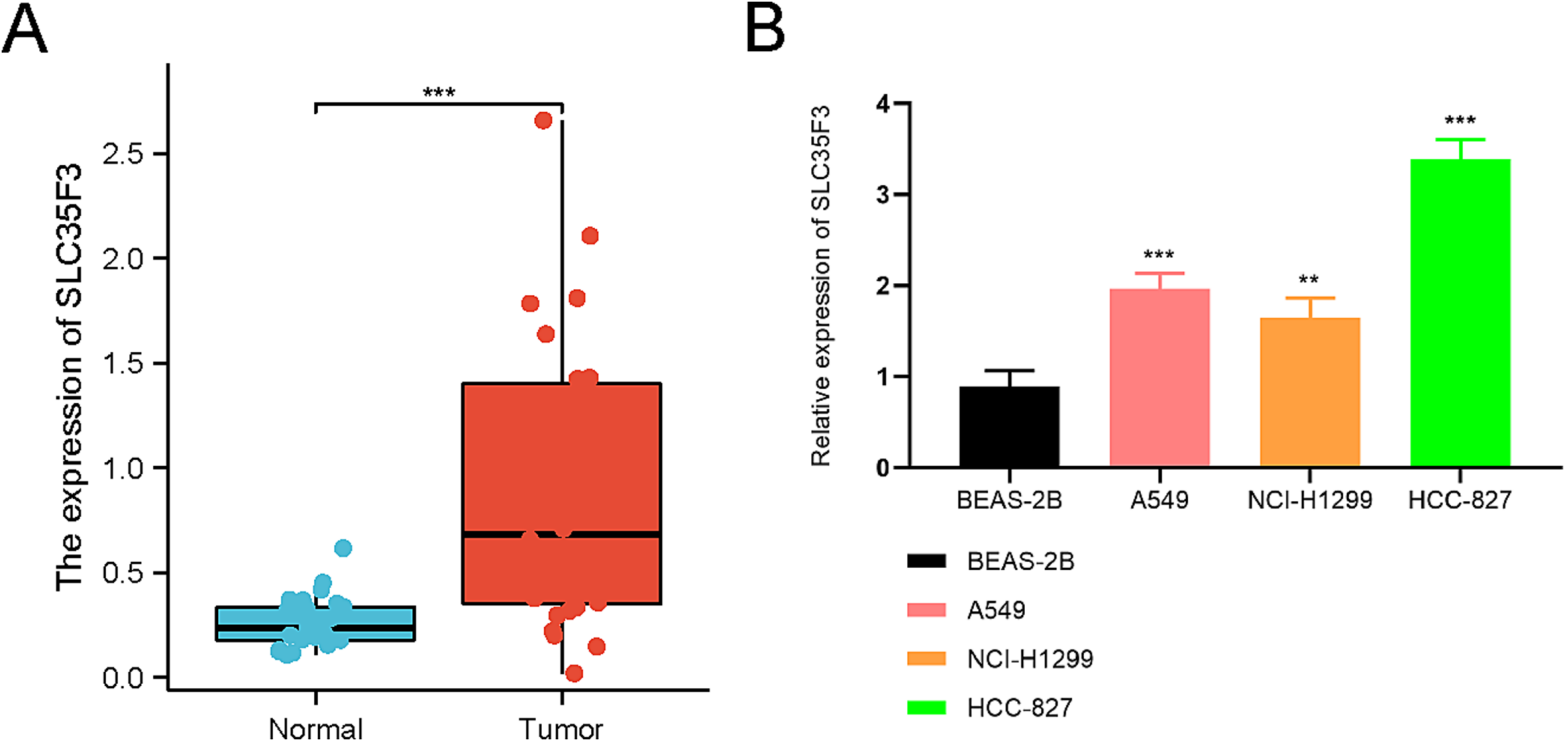
Expression of SLC35F3 in LUAD tissues and cell lines. (A) Expression of SLC35F3 in LUAD tissues. (B) Expression of SLC35F3 in Beas‐2B, A549, NCI‐H1299, and HCC827. ** *p* < 0.01; *** *p* < 0.001.

The data depicted in Figure 8A indicate a significant increase in SLC35F3 expression in LUAD tissues relative to normal lung tissues (*p* < 0.001). Furthermore, SLC35F3 expression was notably higher in A549 cells compared with Beas‐2B cells (*p* < 0.001) and in NCI‐H1299 cells compared with Beas‐2B cells (*p* = 0.004) (Figure 8B). Additionally, SLC35F3 expression was significantly elevated in HCC827 cells compared with Beas‐2B cells (*p* < 0.001) (Figure 8B).

## 4 Discussion

The pathogenesis of LUAD is closely linked to variations in gene expression levels. Elevated levels of SAPG5 in LUAD are indicative of a negative prognosis and increased immune infiltration [30]. Conversely, decreased levels of RASGRP2 in LUAD have been linked to a poorer prognosis and increased immune infiltration [31]. Similarly, the upregulation of PBK in LUAD is significantly associated with an unfavorable prognosis and immune infiltration [2]. High expression of TEDC2 in LUAD is similarly linked to poor prognosis and immune infiltration [32]. It is imperative to investigate novel molecular markers to gain a comprehensive understanding of the prognostic, diagnosis, and treatment in LUAD.

The expression of SLC35F3 in PTC is significantly upregulated [13]. This study identified a significant difference in the expression of SLC35F3 between LUAD tissues and normal tissues (*p* < 0.001). Gender was found to be associated with the expression of SLC35F3 (*p* < 0.001). Furthermore, the expression of SLC35F3 was significantly correlated with poor OS (*p* = 0.020) in patients with LUAD. Furthermore, a distinct and independent association was observed between SLC35F3 expression (*p* = 0.032) and OS in LUAD patients. SLC35F3 was aberrantly expressed in some tumors. Whether SLC35F3 can be a marker for multiple types of cancer will depend on a joint study by multiple teams, and we may conduct this joint study in the future.

Presently, there is a dearth of research on the SLC35F3 regulatory pathway. However, this study elucidated the association between SLC35F3 and various pathways, including cell cycle and more. SLE has been linked to the carcinogenic process [33]. The presence of CNVs related to starch and sucrose metabolism genes may contribute to the development of lung squamous cell carcinoma (LUSC) [34]. The cytochrome P450 enzyme is responsible for the biological transformation of drugs, xenobiotics, and endogenous substances and is involved in the development of cancer [35]. Type I epithelioid malignant pleural mesothelioma (eMPMs) are associated with steroid hormone biosynthesis [36]. The sphk1/S1PR3/pbx1 axis plays a crucial role in regulating the cell cycle of NSCLC, and targeting sphk1 could offer therapeutic benefits in tumor treatment [37]. The specific molecular mechanism of SLC35F3‐mediated LUAD needs further investigation.

The significance of TMB as a biomarker in tumor immunotherapy has been well established [38]. Our study identified a negative association between SLC35F3 expression and TMB in LUAD. Additional research is required to elucidate the specific mechanism that underlies the correlation between SLC35F3 and TMB in LUAD.

The exact nature of the connection between SLC35F3 and the tumor microenvironment (TME) is still ambiguous. Given the significant therapeutic implications of TME in the management of LUAD, this study aims to investigate the association between SLC35F3 expression and TME. The results of our study demonstrate a significant correlation between the expression of SLC35F3 and the infiltration levels of various immune cell subsets. These findings suggest that further investigation into the potential of SLC35F3 as an immunotherapeutic target in LUAD is warranted.

There are currently no research reports on the relationship between SLC35F3 and drug sensitivity. In the present study, we found that the expression of SLC35F3 was negatively correlated with sensitivity to drugs such as 2‐bromo‐, holacanthone, gw694234a, and more. Understanding this can help doctors avoid drugs that may not be effective and instead look for other treatments that may be more effective. By studying the relationship between SLC35F3 and drug sensitivity, it could also provide potential targets for the development of new drugs.

In this study, SLC35F3 was investigated for its role in LUAD. However, it is subject to certain limitations. First, the study relied predominantly on data analysis from publicly available databases. The lack of validation of real‐world patient data in this study may affect the clinical applicability of the results. We will include patients with real‐world LUAD to validate the clinical significance of SLC35F3 in the future. Second, the present study is mainly based on bioinformatics analyses, an approach that, although informative, may need to be further validated by experimental methods to understand the molecular mechanisms of SLC35F3 in LUAD. Future research will incorporate additional experimental approaches to understand the molecular mechanisms underlying LUAD mediated by SLC35F3. Third, drug sensitivity analyses may require additional experimental validation to determine the exact relationship between SLC35F3 expression and drug response.

## 5 Conclusions

SLC35F3 exhibited a marked upregulation in expression levels in LUAD and was significantly correlated with poor OS. SLC35F3 has been implicated in the advancement of LUAD through various pathways, such as the cell cycle and more. SLC35F3 has been linked to immune infiltration and TMB. The expression of SLC35F3 showed significant negative correlations with 2‐bromo‐, holacanthone, 4‐thiazine, and more in LUAD. The findings of this study suggest that SLC35F3 has potential as a prognostic biomarker and immunotherapy target for LUAD.

## Author Contributions

Conception: Yiwang Ye and Yuancai Xie; Funding acquisition: Yiwang Ye; Methodology: Yiwang Ye and Yuancai Xie; Data curation and analysis: Yiwang Ye, Feihu Long, Wei Yue, Zichun Wei, Jianyi Yang; Experimental validation: Yiwang Ye; Original manuscript writing, Yiwang Ye; Writing – review and editing: all authors.

## Ethics Statement

The research adhered to the principles outlined in the Declaration of Helsinki and obtained exemption from the Peking University Shenzhen Hospital Ethical Review Committee.

## Conflicts of Interest

The authors declare no conflicts of interest.

## Supporting information


**Table S1** A correlation between SLC35F3 expression and clinical features in LUAD patients.
**Table S2.** Logistic analysis of the correlation between SLC35F3 expression and clinical characteristics.
**Table S3.** OS and clinical characteristics of LUAD patients with univariate and multivariate analysis (Cox regression).
**Table S4.** Drug sensitivity analysis of SLC35F3.

## Data Availability

This article encompasses the entirety of data produced or scrutinized throughout the course of the study.
